# Thiol Metabolism and Volatile Metabolome of *Clostridioides difficile*

**DOI:** 10.3389/fmicb.2022.864587

**Published:** 2022-06-16

**Authors:** Peter Biwer, Meina Neumann-Schaal, Petra Henke, Dieter Jahn, Stefan Schulz

**Affiliations:** ^1^Institute of Organic Chemistry, Technische Universität Braunschweig, Braunschweig, Germany; ^2^Department of Metabolomics, Leibniz Institute DSMZ-German Collection of Microorganisms and Cell Cultures, Braunschweig, Germany; ^3^Braunschweig Integrated Centre of Systems Biology, BRICS, Braunschweig, Germany; ^4^Institute of Microbiology, Technische Universität Braunschweig, Braunschweig, Germany

**Keywords:** *Clostridium difficile*, thiols, disulfides, sulfur metabolism, gas chromatography/mass spectrometry, cysteine

## Abstract

*Clostridioides difficile* (previously *Clostridium difficile*) causes life-threatening gut infections. The central metabolism of the bacterium is strongly influencing toxin production and consequently the infection progress. In this context, the composition and potential origin of the volatile metabolome was investigated, showing a large number of sulfur-containing volatile metabolites. Gas chromatography/mass spectrometry (GC/MS)-based headspace analyses of growing *C. difficile* 630Δ*erm* cultures identified 105 mainly sulfur-containing compounds responsible of the typical *C. difficile* odor. Major components were identified to be 2-methyl-1-propanol, 2-methyl-1-propanethiol, 2-methyl-1-butanethiol, 4-methyl-1-pentanethiol, and as well as their disulfides. Structurally identified were 64 sulfur containing volatiles. In order to determine their biosynthetic origin, the concentrations of the sulfur-containing amino acids methionine and cysteine were varied in the growth medium. The changes observed in the volatile metabolome profile indicated that cysteine plays an essential role in the formation of the sulfur-containing volatiles. We propose that disulfides are derived from cysteine *via* formation of cystathionine analogs, which lead to corresponding thiols. These thiols may then be oxidized to disulfides. Moreover, methionine may contribute to the formation of short-chain disulfides through integration of methanethiol into the disulfide biosynthesis. In summary, the causative agents of the typical *C. difficile* odor were identified and first hypotheses for their biosynthesis were proposed.

## Introduction

*Clostridioides difficile* (previously *Clostridium difficile*) is a major nosocomial human pathogen with a significant number of community-acquired infections (Knight et al., 2015; Lessa et al., 2015). It can be isolated from mammals, various birds and reptiles, as well as from the environment and food (Hensgens et al., 2012). The transmissibility of the pathogen is increased by the formation of highly resistant spores, which can survive various stress conditions and persist in the environment for months or even up to years (Barra-Carrasco and Paredes-Sabja, 2014). Symptoms of *C. difficile* infections (CDI) range from relatively mild diarrhea over pseudomembranous colitis to sepsis with high morbidity and mortality (Nanwa et al., 2015). To current knowledge, these symptoms are caused by the toxins A (TcdA) and B (TcdB) which lead to extensive intestinal damage and pathology (Carter et al., 2015). Some *C. difficile* isolates also produce a binary toxin (CDT; Aktories et al., 2018).

For clostridial growth and toxin mediated pathogenicity, the metabolic network and the nutritional status in the environment play an important role. Its favored energy sources, the amino acids proline and the sulfur-containing cysteine reduce toxin production independent of the used growth medium and tested strain (Karlsson et al., 2000). Cysteine-dependent toxin gene regulation appears to be related to products of the cysteine degradation, mainly pyruvate, lactate, and probably sulfide (Dubois et al., 2016; Gu et al., 2018b; Hofmann et al., 2021). The addition of a mixture of seven amino acids (glycine, isoleucine, leucine, methionine, threonine, tryptophan, and valine) as well as the vitamin biotin led to similar effects (Karlsson et al., 1999, 2000, 2008). A major process for energy production by *C. difficile* is the Stickland reaction, a coupled fermentation of amino acids (Stickland, 1934). After initial enzymatic deamination, resulting the 2-ketoacids are either oxidized or reduced in a coupled reaction to their corresponding organic acids *via* coenzyme A-activated intermediates. Energy is conserved by substrate-level phosphorylation and electron-bifurcating enzymes coupled to the Rnf-complex (Aboulnaga et al., 2013; Buckel and Thauer, 2013; Dannheim et al., 2017a; Neumann-Schaal et al., 2019). Depending on the amino acid, one amino acid is oxidized while up to two others are reduced. Certain amino acids like proline and glycine are metabolized *via* modified Stickland pathways (Stadtman, 1966; Jackson et al., 2006). Alanine, cysteine, and serine are metabolized *via* the central carbon metabolism and enter it *via* pyruvate. Threonine is degraded *via* acetaldehyde and glycine to acetyl-CoA, or *via* 2-oxobutanoate to propanoyl-CoA (Fonknechten et al., 2010). The products of the central carbon metabolism-associated fermentation are butanoate and pentanoate and further propanoate, lactate, and acetate (Aboulnaga et al., 2013; Dannheim et al., 2017b; Hofmann et al., 2021). While in earlier growth phases the exometabolome is dominated by broad range of organic acids, corresponding alcohols can be detected in later growth stages, specifically when intracellular coenzyme A pools are depleted (Hofmann et al., 2018).

*Clostridioides difficile* cultures possess very distinctive odors that can be attributed to a set of volatile organic compounds (VOCs). Odor-based determination of CDI with trained dogs have previously been reported (Charles et al., 2019). Furthermore, identification methods utilizing gas chromatography/mass spectrometry (GC/MS) were investigated as another potential tool for rapid diagnosis of CDI. Through these efforts, several compound classes like amines, organic acids, alcohols, thiols, and disulfides were identified among others (Pons et al., 1985; Garner et al., 2007; Rees et al., 2016). Sulfur-containing VOCs occur widely spread across bacteria (Schulz and Dickschat, 2007; Schulz et al., 2020; Weisskopf et al., 2021) equipping its emitters with different bioactivities (Netzker et al., 2020; Schulz et al., 2020; Weisskopf et al., 2021) in interactions with animals (Popova et al., 2014), plants (Huang et al., 2012; Meldau et al., 2013), fungi (Fernando et al., 2005; De Vrieze et al., 2015), other bacteria (Dandurishvili et al., 2011; Groenhagen et al., 2013; Tyc et al., 2015) and in health (Walker and Schmitt-Kopplin, 2021). This obvious importance of volatile substances for the biology of *C. difficile* in combination with a lack of systematic investigations of these compounds prompted us to start with a chemical inventory of VOCs and first physiological studies for their biochemical origins.

Thus, the rationale of our approach was to investigate the VOC metabolome of the widely used and well-characterized model strain *C. difficile* 630Δ*erm* (DSM 28645) *via* direct headspace extraction and GC/MS analysis under anaerobic conditions and chemical synthesis of candidate compounds with an emphasis on thiols and disulfides. Furthermore, we explored the influence of sulfur-containing amino acids cysteine and methionine on the biosynthesis of corresponding VOCs.

## Experimental

### Cultivation of Bacteria

Studies were performed with *Clostridioides difficile* 630Δ*erm* (DSM 28645; Hussain et al., 2005) obtained from the German Collection of Microorganisms and Cell Cultures (DSMZ, Braunschweig, Germany, recent genome data published by Dannheim et al., 2017b). *Clostridioides difficile* 630Δ*erm* is a spontaneous erythromycin-sensitive mutant of the isolate 630 (Wüst and Hardegger, 1983) and was originally isolated as erythromycin-, tetracycline-, and clindamycin-resistant strain in a clinical environment in Switzerland. It contains the PaLoc encoding for the two major toxins, TcdA and TcdB. Several studied on metabolic properties (incl. cysteine metabolism), physiology and spore formation have been published (e.g., Gu et al., 2018a,b; Hofmann et al., 2018, 2021; Wetzel and McBride. 2020; Brauer et al., 2021). Main cultures were cultivated in medium CDMM as described earlier (Neumann-Schaal et al., 2015). Casamino acids were obtained from Merck (Darmstadt, Germany, lot number VM692545514) and the exact amino acid content of this lot has been quantified in a previous study (Will et al., 2017). Casamino acids contain all proteinogenic amino acids except glutamine, asparagine, cysteine, and tryptophan. The latter two are required for growth of *C. difficile*. To prepare the final CDMM medium, the amino acid mixture was further supplemented with 0.5 g/L cysteine and 0.1 g/L tryptophan resulting in 0.5 g/L cysteine (as added separately) and 0.12 g/L methionine (originating from the casamino acids) as sulfur-containing amino acids and potential sources for thiol formation by *C. difficile* (called CDMM in the text). Additionally, the experiments were performed with CDMM medium containing less (0.1 g/L, 0.8 mmol/L final concentration, CDMM-C) or more (2.0 g/L, 16.5 mmol/L final concentration, CDMM+C) cysteine, and with medium containing an increased amount of methionine (+1 g/L 7.5 mmol/L final concentration, CDMM+M) compared to the CDMM described above. Cells were transferred twice with a dilution of 1:200 in CDMM prior to inoculation of the main culture. Main cultures (100 ml culture volume in 125 ml Afnor bottles, chlorobutyl septa, Zscheile & Klinger, Hamburg, Germany) were inoculated with a dilution of 1:100 of an actively growing preculture (OD_600nm_ ~ 0.5). The cultures were incubated at 37°C for 24 h to the late stationary phase. All cultivations were performed as three independent biological replicates.

### Sampling and Analysis of the Volatile Metabolome

Three independent biological cultures of *C. difficile* grown on media containing high (CDMM+C) or reduced quantities (CDMM-C) of cysteine as well as media with increased amounts of methionine (CDMM+M) were used to investigate relative changes in VOC concentrations during cultivation. The standard CDMM medium, also analyzed three times, served as reference point and background control for medium derived VOCs. A medium analysis of CDMM without *C. difficile* served as control to exclude compounds originating from the medium. Headspace extracts were obtained by a nitrogen flow (0.1 L/min) through the anaerobic liquid bacterial culture in its late stationary growth phase and transfer to a thermal desorption tube filled with an absorbent (Tenax TA Tube; GERSTEL, Mülheim an der Ruhr, Germany) for 2 h. The trapped compounds were analyzed by GC/MS using a thermal desorption unit (TDU), cooled injection system (CIS), and a MultiPurposeSampler (MPS) autosampler (GERSTEL, Mülheim an der Ruhr, Germany) connected to an Agilent 7890B gas chromatograph. The gas chromatograph was equipped with a HP-5 MS fused silica capillary column (30 m, 0.25 i. d., 0.25 μm film, Hewlett-Packard, Wilmington, United States) connected to an Agilent 5977A mass-selective detector. Conditions: transfer line 300°C, electron energy 70 eV. Thermal desorption: 30°C, increasing at 60°C/min to 280°C, 10 min isothermal. Cooled injection: −150°C, increasing at 12°C/s to 300°C, 3 min isothermal; 0.6 s splitless transfer. Gas chromatographic method: 50°C, 5 min isothermal, increasing at 5°C/min to 320°C; operated in splitless mode. Helium was used as carrier gas with a flow of 1.2 ml/min. Linear GC retention indices (*RI*) were determined from a homologous series of *n*-alkanes (C_8_–C_30_).

### Data Analysis and Disulfide Microreactions

The bacterial volatile compounds were identified by comparison of their mass spectra and retention indices with data obtained from mass spectral databases, commercially available or synthesized authentic samples and literature values. Identical GC and MS data verified the compound structure. Compounds that were found in at least two of three replicates were included in this study. Media blanks were analyzed separately, and its constituents were subtracted from the obtained VOC list. Relative compound quantities were calculated as ratio between mean integrated signal found in the respective test medium and mean integrated signal found in the reference medium. When a compound was not detected during growth in the reference CDMM medium, the lowest detected mean integrated signal was used as reference point. Relative compound quantities are visualized as fold changes. Significant changes in compound concentrations were calculated by Wilcoxon-Mann–Whitney test including a Benjamini-Hochberg correction using TigrMev software (version 4.6.2, Saeed et al., 2003). Both, *p*-value and adjusted *p*-value are supplied in Supplementary Table S1.

### General Synthetic Method for Synthesis of Disulfides

Two disulfides (0.1 mmol each) were mixed with Et_3_N (0.2 mmol) and DMF (100 μl) in a 1.5 ml vial. The vial was sealed and placed in a sonication bath at 40°C. After 45 min of sonication H_2_O (250 μl) and diethyl ether (250 μl) were added (Ruano et al., 2008). The organic phase was separated, dried with NaCl, and diluted 1:50. Around 1 μl of the solution was injected into the GC/MS system.

## Results

The metabolism of *C. difficile* plays a major role in the pathogenicity of the organism (Neumann-Schaal et al., 2019) and everyone investigating this organism is familiar with its unique odor, which is caused by various sulfur-containing VOCs. However, the nature and function of volatile metabolic products of *C. difficile* are mainly unknown. In a first step an inventory of these volatile substances was established by isolating them from the headspace of a *C. difficile* culture and determining their chemical structure using GC/MS. Thus, a detailed structural analysis of the volatile compounds of *C. difficile* 630Δ*erm* emphasizing thiols and disulfides was performed. Unknown compounds were identified by analyzing their mass spectra and comparing their data with those of chemically synthesized potential candidate compounds. Culture conditions concerning the sulfur-containing amino acids methionine and cysteine were varied and the VOC amounts determined in order to establish the origin of the sulfur groups of the various found VOCs. Because these amino acids likely are the biosynthetic sources of sulfur, an influence on the volatile production was anticipated, leading to an insight into the biosynthetic pathways associated with VOC production in *C. difficile*. Media containing high (CDMM+C) or reduced quantities (CDMM-C) of cysteine as well as media with increased amounts of methionine (CDMM+M) were used to investigate relative changes in VOC concentrations during cultivation.

### Identification of Volatiles Released by *Clostridioides difficile* 630Δ*erm*

The VOCs emitted by *C. difficile* 630Δ*erm* under strictly anaerobic conditions were trapped on an adsorbent and directly analyzed by GC/MS using thermodesorption. This headspace method allowed sensitive direct analysis of the emitted compounds. The analysis of the headspace of a CDMM culture without bacteria served as background control and revealed a number of volatile compounds to be released by the medium, which were excluded from further analysis.

**Figure 1:**
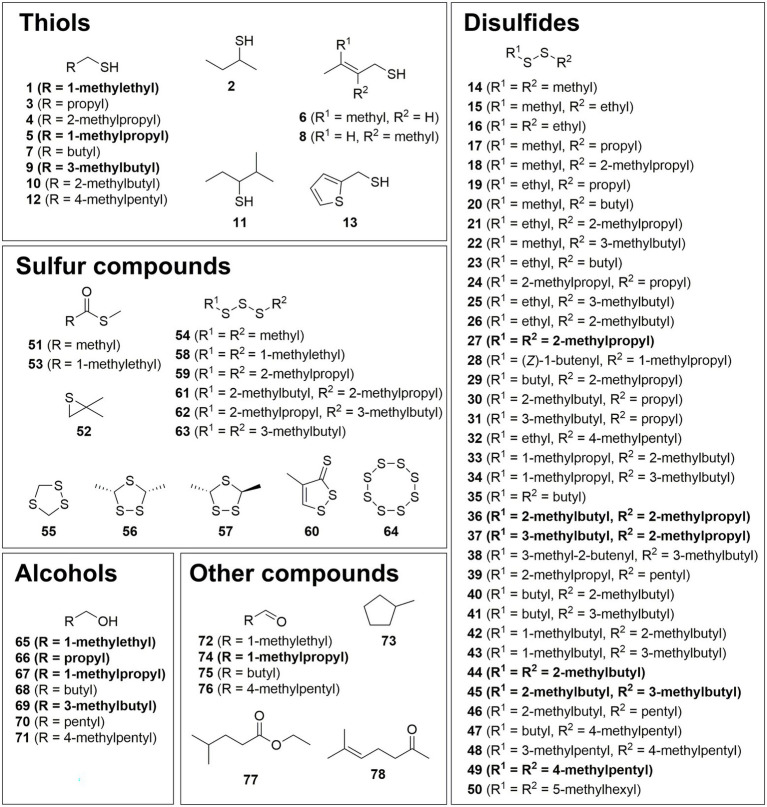
Structures of identified compounds grouped into thiols, disulfides, sulfur compounds, alcohols, and other compounds. Major constituents are shown in bold.

**Figure 2:**
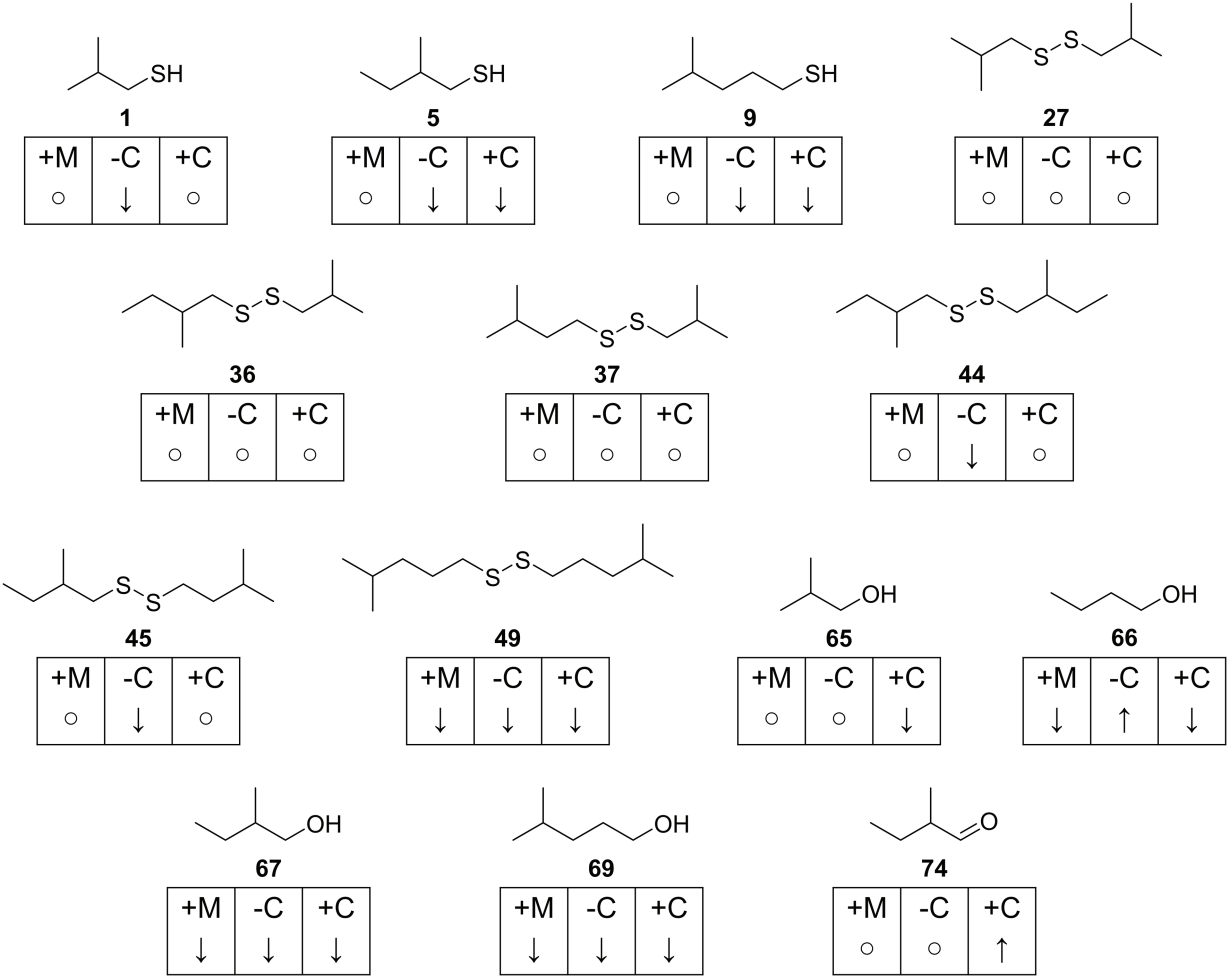
Overview of most abundant compounds found in *Clostridioides difficile* 630Δ*erm*. Concentration changes for each compound are indicated with ↑ (increase), ↓ (decrease or not detected) and ○ (fold change < 1.5) and given for each test medium in reference to CDMM. +M: CDMM+M; -C: CDMM-C; +C: CDMM+C.

**Figure 3:**
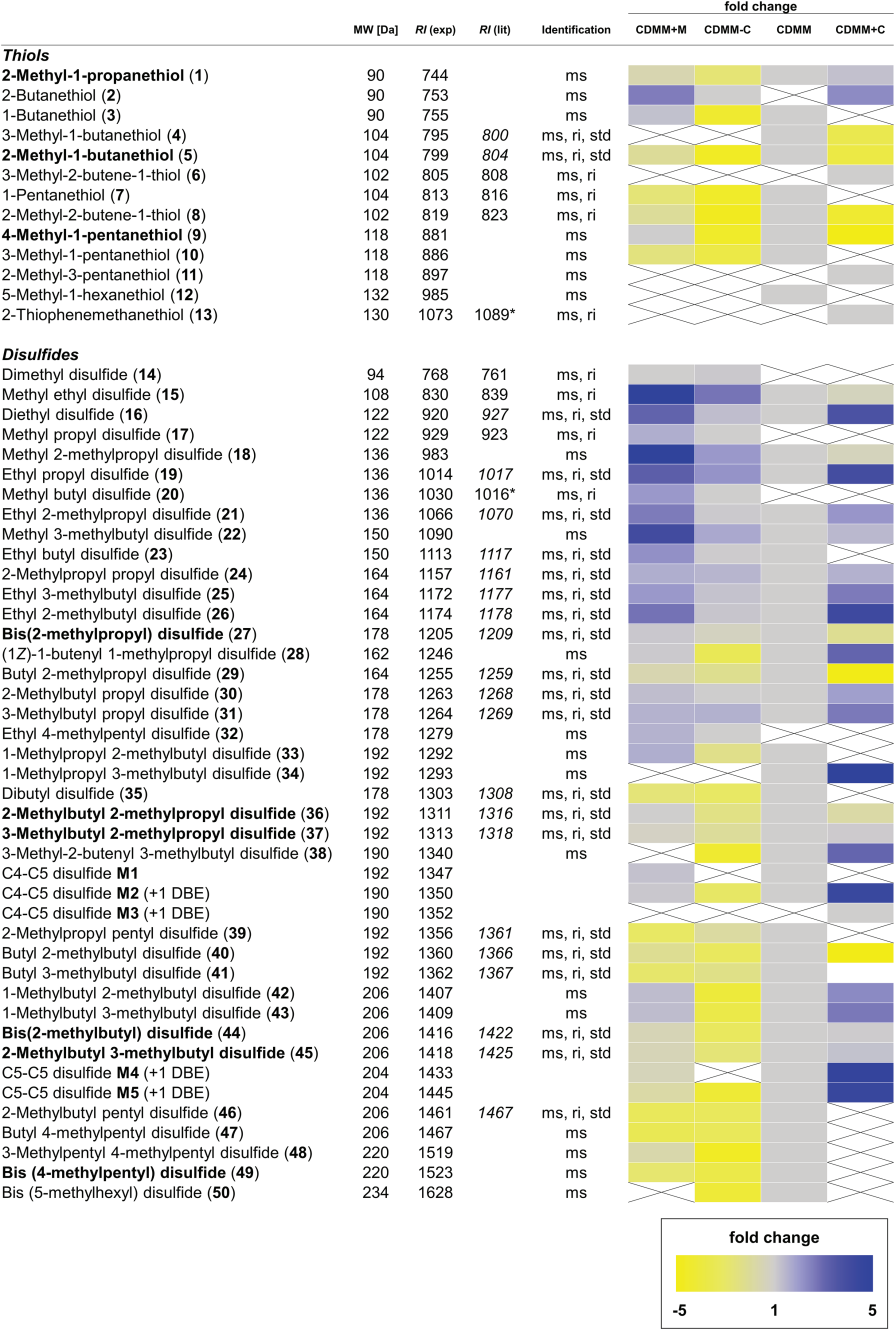
Overview of volatile thiols and disulfides found in *Clostridioides difficile* 630Δ*erm*. Compounds annotated in bold are major components of the volatile bouquet. The molecular weight (MW) is given in Da. Compound identification was based on comparison of spectra with those of data bases and mass spectrometric fragmentation (ms), comparison of retention index to published values on the same or similar GC phases (ri), as well as comparison to commercially available or synthetic reference compounds (std). Retention indices from our own database are shown in italic. Retention indices determined on a GC phases related to but not identical to the HP5-MS phase used are marked with an asterisk. Fold changes of integrated signals from CDMM+M, CDMM-C, and CDMM+C were referenced to CDMM. When one compound was not found in reference CDMM, the lowest integrated signal detected was used as referencing point. Muted yellow squares represent a decrease fold change of −1.1 to −5.0 and bright yellow squares decrease fold changes <− 5.0. Light blue squares represent an increase fold change of 1.1–5.0 and dark blue squares >5.0. Gray squares show fold changes between 0.9 and 1.1. Crossed, white squares represent compounds below the detection limit.

**Figure 4:**
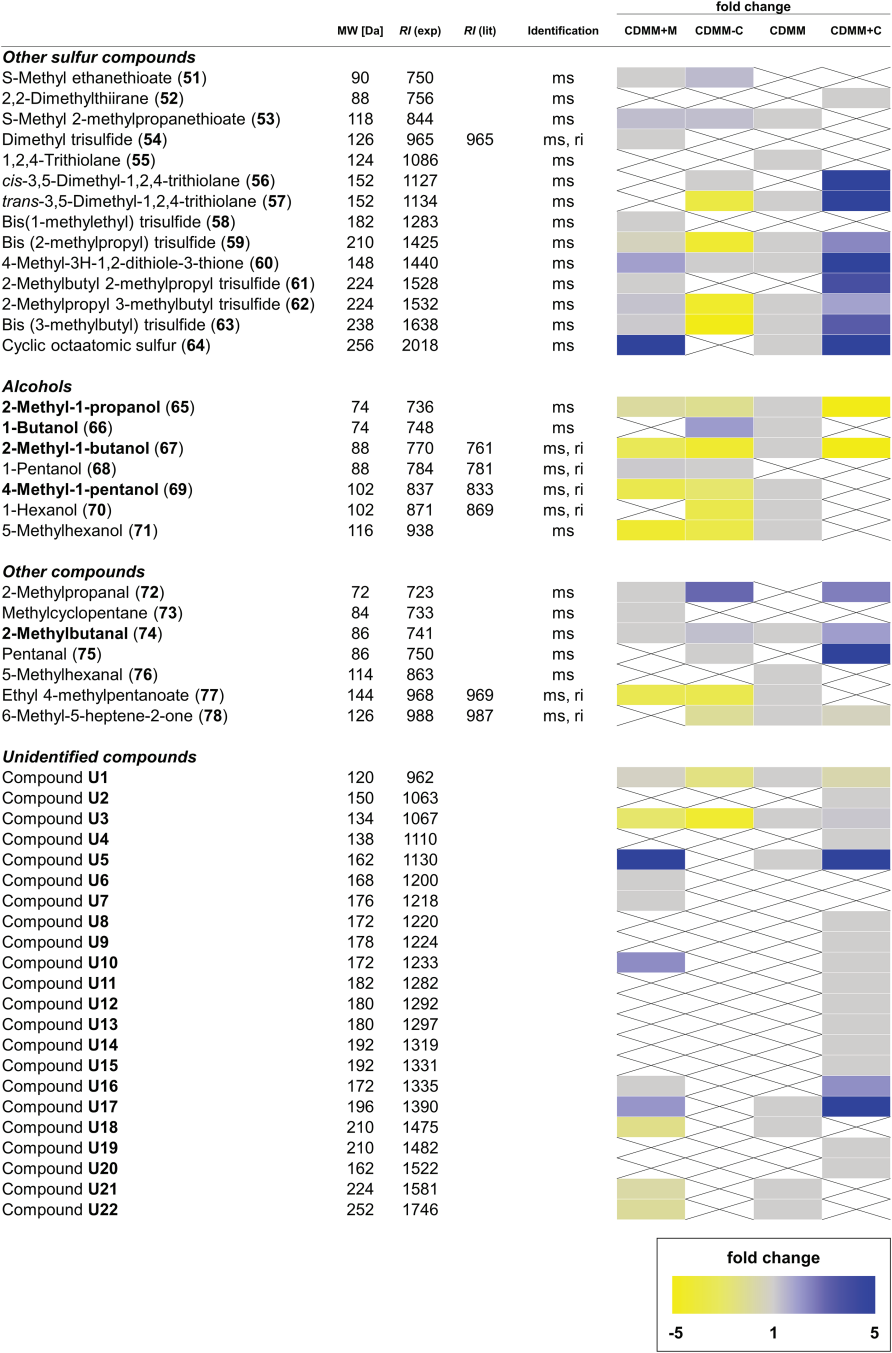
Overview of other sulfur-containing and non-sulfur-containing volatile organic compounds (VOCs), as well as alcohols and unidentified compounds found in *Clostridioides difficile* 630Δ*erm*. For details see Figure 3.

The VOCs were identified by comparison of their mass spectra and gas chromatographic retention indices with data from mass spectral databases, authentic samples, and literature values (Figures 1–4; Supplementary Table S1). Furthermore, specific analysis of the mass spectral fragmentation revealed the structural identity of unknown compounds. The VOC composition of CDMM cultures contained a number of asymmetrical and symmetrical disulfides that showed unknown mass spectra. Therefore, candidate compounds needed to be synthesized for identification. To reduce the synthetic effort, these disulfides were synthesized in microreactions from two commercially available thiols using a method adapted from Ruano et al. (2008) (Supplementary Figure S3). The crude products, resulting in three possible disulfides, were analyzed by GC/MS to determine their retention indices and EI mass spectra. The synthesized compounds and mass spectral data are listed in Supplementary Table S2. Disulfides that could not be identified through this method were partially identified by their mass spectra, which revealed alkyl side chain sizes and double bond equivalents. Furthermore, a range of trisulfides occurred in low quantities, which structures remained unresolved. They are labeled as unknowns throughout the text. The 14 most abundant compounds released by *C. difficile* 630Δ*erm* were the thiols 2-methyl-1-propanethiol **(1)**, 2-methyl-1-butanethiol **(5)** and 4-methyl-1-pentanethiol **(9)**, the disulfides bis(2-methylpropyl) disulfide **(27)**, 2-methylbutyl 2-methylpropyl disulfide **(36)**, 2-methylbutyl 3-methylpropyl disulfide **(37)**, bis(2-methylbutyl) disulfide **(44)**, 2-methylbutyl 3-methylbutyl disulfide **(45)** and 3-methylpentyl 4-methylpentyl disulfide **(49)**, the related alcohols 2-methyl-1-propanol **(65)**, 1-butanol **(66)**, 2-methyl-1-butanol **(67)**, and 4-methyl-1-pentanol **(69)**, as well as 2-methylbutanal **(74)** (Figure 1). All these compounds shared similar carbon backbones originating from alkyl amino acids. Altogether 71 different compounds were identified with the CDMM medium. The 14 most abundant compounds and their concentration changes in CDMM+M. CDMM-C and CDMM+C are shown in Figure 2.

### Influence of Methionine on the Thiol and Disulfide VOC Profile of *Clostridioides difficile* 630Δ*erm*

When *C. difficile* 630Δ*erm* was offered increased amounts of methionine as additional sulfur source (CDMM+M), the abundance of four thiols changed. 2-Butanethiol **(2)** appeared while 3-methyl-1-butanethiol **(4)** and 5-methyl-1-hexanethiol **(12)** were not detected anymore. 1-Pentanethiol **(7)** concentrations decreased 1.5-fold, while proportions of all other thiols did not change (Fold change < 1.5; Figure 3).

Within disulfides, the highest fold-changes were found among short-chain disulfides. The most prominent cases are dimethyl disulfide **(14)**, methyl propyl disulfide **(17)** and methyl butyl disulfide **(20)** that were found in CDMM+M, but not detectable in CDMM. Abundances of the remaining methyl disulfides (**15**, **18**, and **22**) were increased 4.1–5.1-fold. Disulfides carrying an ethyl group and a C_2_–C_5_ alkyl chain (**16**, **19**, **21**, **23**, and **26**) were detected in 2.2–3.2-fold higher concentrations in CDMM+M and ethyl 4-methylpentyl disulfide **(32)** occurred. The abundances of the majority of disulfides with C_3_ or longer chains did not change (fold change < 1.5), with the exception of 2-methylpropyl propyl disulfide **24**, while disulfides **34**, **38**, and **50** were not detected (Figure 3). Disulfides carrying a methyl group and to a lesser degree those with an ethyl group increased, while the larger disulfides or trisulfides did not change markedly.

Additionally, the increased methionine content resulted in changes among other sulfur compounds, pointing toward an increased sulfur metabolism, exemplified by the 5.1-fold increase of cyclic octameric sulfur **(64)**. *S*-Methyl ethanethioate **(51)**, as well as trisulfides **54**, **58**, and **61** were only produced in CDMM+M, while CDMM compounds 1,2,4-trithiolane **(55)** and *trans*-3,5-dimethyl-1,2,4-trithiolane **(57)** were not detected. Abundances of *S*-methyl 2-methylpropanethioate **(53)**, 4-methyl-3*H*-1,2-dithiole-3-thione **(60)** and the larger trisulfides **59**, **62**, and **63** were not altered. In most cases, the concentrations of alcohols decreased in CDMM+M except for 1-pentanol **(68)**, which was found in this medium, but not in CDMM. Other medium specific compounds specific include 2-methylpropanal **(72)** and methylcyclopentane **(73)**, while the CDMM compounds 5-methylhexanal **(76)** and 6-methyl-5-heptene-2-one **(78)** were lacking. Eleven unidentified compounds were found in the CDMM+M cultures, four of them (**U6**, **U7**, **U10**, and **U16**) not present in CDMM, while the rest remained unchanged compared to CDMM (Figure 4).

The results indicate that methionine in *C. difficile* 630Δ*erm* functions as a source of methanethiol, as has been shown for a variety of other bacteria. Methanethiol, difficult to detect by headspace GC/MS method due to its very weak interaction with adsorbents, serves as a precursor for disulfide formation, as indicated here by the increased methyl alkyl disulfide formation. Thiols are sensitive to oxidation, methanethiol reacts under aerobic conditions easily to form dimethyl disulfide. Under the anaerobic conditions of *C. difficile* methanethiol might react with or without enzymatic involvement with other sulfides to disulfides. This might explain the high levels of methyl alkyl disulfides found with the CDMM+M medium.

### Influence of Cysteine on the Thiol and Disulfide VOC Profile of *Clostridioides difficile* 630Δ*erm*

Cysteine is another important amino acid involved sulfur VOC biosynthesis. Decreasing or increasing the cysteine content of CDMM led to changes in VOC formation. With CDMM-C a decline in volatile thiols was observed. Seven thiols (**1**, **3**, **5**, **7**, **8**, **9**, and **10**) were produced in 1.5–3.9-fold lower quantities and two (**4** and **12**) were not detected compared to CDMM. 2-Butanethiol **(2)** was the only compound that was found at low and high cysteine concentrations, but not detected in CDMM. Additional thiol compounds 3-methyl-2-butene-1-thiol **(6)**, 2-methyl-3-pentanethiol **(11)** and 2-thiophenemethanethiol **(13)** were observed at high cysteine concentrations (CDMM+C), while the majority of remaining thiol concentrations was lowered (Figure 3).

Within the disulfides, shifts in the VOC profile were observed. When cysteine amounts were lowered, concentrations of short-chain disulfides (**15**, **16**, **18**, **19**, and **22**) rose. The majority of medium and long-chain disulfide concentrations remained mostly stable and rarely occurred with lower amounts (disulfides M4, M5, 34, 38, and 50). Disulfides **14**, **17**, **20**, and **32** were only produced in CDMM-C, but not produced in reference CDMM and at higher cysteine levels. When cysteine amounts were increased, a reduction in the overall number of volatile disulfides was observed. Out of 38 disulfides detected in CDMM, 11 were not detected (**23**, **33**, **35**, **39**, **41**, **46**–**50**, and M1) and concentrations of butyl 2-methylpropyl disulfide **(29)** and butyl 2-methylbutyl disulfide **(40)** decreased 10-fold in CDMM+C. In contrast, an increase was found for 12 compounds (**16**, **19**, **21**, **24**, **26**, **28**, **30**, **31**, **38**, **M2**, **M4**, and **M5**) with fold changes ranging from 1.6 to 4.9. Moreover, production of one unique compound (C4-C5 disulfide **M3**) was observed under these conditions and concentrations of 12 other compounds did not change (fold changes < 1.5; Figure 4). The structural variety in disulfides remained mostly the same with CDMM-C, although concentrations were reduced, while increase of cysteine led to reduced structural variety, but generally in higher quantities of disulfides.

Variations depending on cysteine content were also observed for other sulfur-containing compounds. At low levels of cysteine, *S*-methyl ethanethioate **(51)** and *cis*-3,5-dimethyl-1,2,4-trithiolane **(56)** were detected, which were not detected in CDMM. While concentrations of *S*-methyl 2-methylpropanethioate **(53)**, *trans*-3,5-dimethyl-1,2,4-trithiolane **(57)** and 4-methyl-3*H*-1,2-dithiole-3-thione **(60)** did not change (fold change < 1.5), no 1,2,4-trithiolane **(55)** and sulfur **(64)** were found in CDMM-C. At high cysteine levels, 2,2-dimethylthiirane **(52)** and *cis*-3,5-dimethyl-1,2,4-trithiolane **(56)** were newly formed species. Moreover, under these growth conditions **53** was not detected, whereas the abundances of **57**, 4-methyl-3*H*-1,2-dithiole-3-thione **(60)** and sulfur **(64)** rose 9.8, 6.7, and 20-fold, respectively (Figure 4).

Additionally, cysteine also seems to influence the formation of volatile alcohols. When the cysteine supply was restricted, 1-butanol **(66)** and 1-pentanol **(68)** productions were increased, while those of 4-methyl-1-pentanol **(69)**, 1-hexanol **(70)** and 5-methyl-1-hexanol **(71)** were decreased 1.6–2.2-fold. Under high levels of cysteine, the alcohol metabolism was almost depleted, leaving only 2-methyl-1-propanol **(65)** and 2-methyl-1-butanol **(67)** detectable, but with concentrations lowered 11.5 and 5.5-fold compared to CDMM. The aldehyde content was variable, with **72** and **75** detectable both in CDMM+C and CDMM-C, whereas **76** was not detected, Concentrations of 2-methylbutanal **(74)** remained stable at low and medium cysteine levels but rose 1.9-fold at high levels. Production of the terpenoid 6-methyl-5-hepten-2-one **(78)** was independent of cysteine concentration, while the concentration maximum of the only alkyl ester detected, ethyl 4-methylpentanoate **(77)**, was reached in CDMM, decreased 2-fold in CDMM-C, but was not detected in CDMM+C (Figure 4).

From these results, we concluded that cysteine directly feeds into the volatile production pathway of *C. difficile* 630Δ*erm*. Thus, addition of cysteine resulted in the formation of numerous thiols and disulfides, indicating that cysteine acts as sulfur source in sulfur volatiles. While decreasing amounts of cysteine resulted in overall lower levels of sulfur volatiles, the increase of cysteine caused a metabolic shift toward concentration of single sulfur volatiles.

In summary, a total of 105 VOCs were detected in the headspace extracts obtained from *C. difficile* 630Δ*erm* under different conditions. Of these, 14 compounds were previously described as *C. difficile* VOCs (Rees et al., 2016) and 78 compounds were structurally characterized (Figures 1–4). Five compounds were partly identified and 22 compounds remained unidentified. Disulfides (42 compounds) and thiols (13 compounds) represented the major compound classes, explaining the well-known odor of *C. difficile* cultures. In addition, 28 compounds of other compound classes were found, alcohols being the most important one. Exemplary total ion chromatograms (TIC) of the analyses are shown in Supplementary Figure S1.

**Figure 5:**
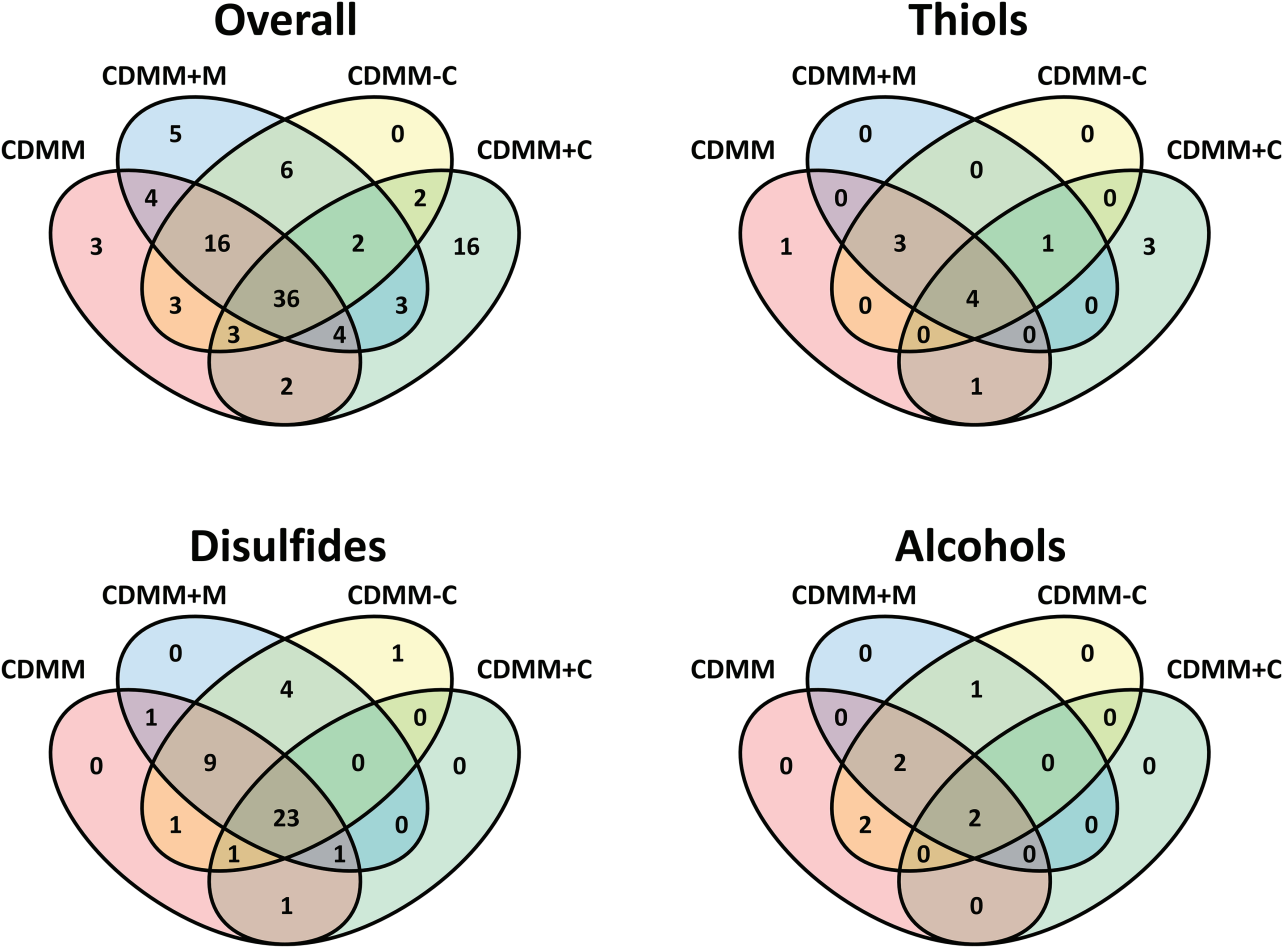
Venn diagrams of volatile organic compounds from *Clostridioides difficile* 630Δ*erm* cultivated in four CDMM derived media. Overall includes all compounds, the other diagrams the respective compound class.

A Venn diagram illustrates the different occurrence of compounds and the high variability depending on individual growth conditions (Figure 5). The reference medium CDMM released 71 VOCs, 36 of which were common to all four media tested (Figure 5). Another 16 compounds were detectable during growth in all media except CDMM+C, while the latter alone had 16 specific VOCs, solely detectable under increased cysteine concentrations. A smaller number of compounds were specific to other combinations as shown in Figure 5. A large number of sulfides and disulfides are constitutively present, while alcohol presence is more variable.

## Discussion

The results showed that the characteristic odor of *C. difficile* is largely due to sulfur containing volatiles. The production of these volatiles is strongly influenced by the addition of methionine and cysteine to the growth medium. Obviously, methionine is serving as a source for methanethiol in this context. Moreover, cysteine not only serves as a source of sulfur, but also influences the VOC composition. Therefore, a direct influence of cysteine on VOC biosynthesis seems very likely.

### Biosynthesis of Thiol and Disulfide VOCs in *Clostridioides difficile* 630Δ*erm*

**Figure 6:**
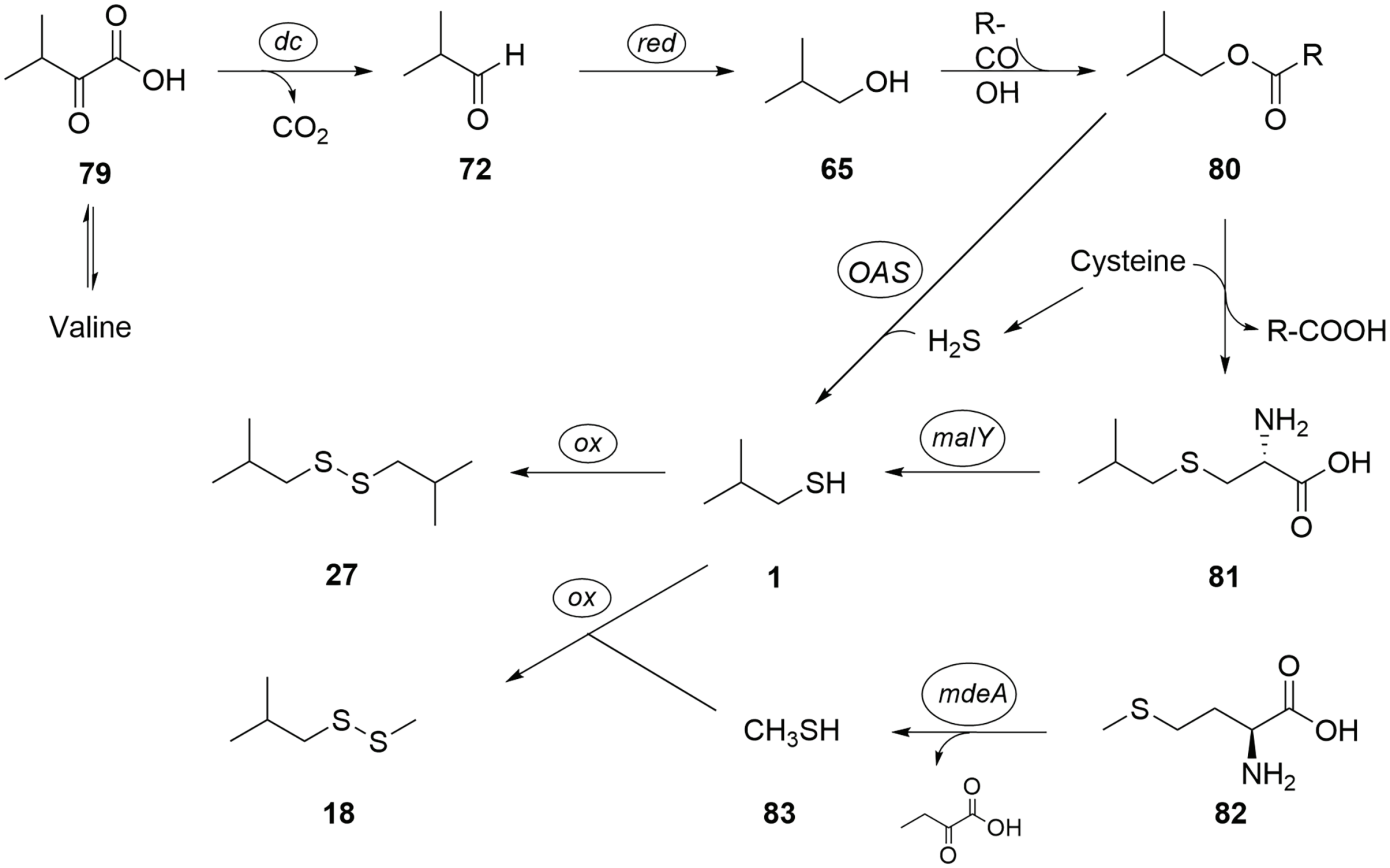
Proposed biosynthetic pathway to thiols and disulfides, exemplified for bis(2-methylpropyl) disulfide **(27)** and methyl 2-methylpropyl disulfide **(18)**. tr: transamination; dc: decarboxylation; OAS: *O*-acetylserine lyase; malY: cystathione lyase; ox: oxidation; and mdeA: methionine γ-lyase.

The biosynthetic pathway toward short-chain thiols has yet to be determined, but a biosynthetic route connected to the corresponding amino acids alanine, valine, leucine, isoleucine, or their respective ketoacids, as well as acetate seems likely. The amino acids are precursors of different organic acids produced by *C. difficile* (Rees et al., 2016) that have the same carbon backbone as many of the sulfur and alcohol compounds. We therefore propose the biosynthetic pathway shown in Figure 6, explaining the formation of 2-methyl-1-propanol **(65)**, methylpropanethiol **(1)** and its dimerization product bis(2-methylpropyl) disulfide **(27)** as well as methyl 2-methylpropyl disulfide **(18)** as an example. 2-Oxo-3-methylbutanoate **(79)**, the transamination product of valine, is converted with loss of CO_2_ into 2-methylbutanal **(72)**, followed by reduction to 2-methyl-1-propanol **(65)**, as has been shown for *Bacillus subtilis* (Li et al., 2011). The sulfur introduction might be realized in a pathway related to cysteine biosynthesis. Therefore, alcohol **65** needs to be activated, e.g., *via* ester **80** in analogy to cysteine biosynthesis from serine that also uses an ester intermediate, *O*-acetylserine (Bogicevic et al., 2016). This step may be realized by secondary activity of CysE (CDIF630erm_01768) or another putative acetyltransferase present in *C. difficile* 630Δ*erm* (e.g., CDIF630erm_00789). With a proper leaving group in place, sulfur introduction might follow directly by reaction with cysteine. The cystationine analog intermediate *S*-methylpropylcysteine **(82)** then potentially releases the sulfide **1** *via* desulfhydrase activity. A cystathionine-β-lyase of the PatB/MalY family (CDIF630erm_03313) with this activity was described by Dubois et al. (2016) for *C. difficile*. Thiol **1** likely then forms disulfide **27** by oxidative dimerization. Although dimerization can occur spontaneously under aerobic conditions, sampling in our case was performed under anaerobic conditions. It might therefore be that an enzymatic process is involved in the dimerization, although the high diversity of disulfides points toward a not very selective process.

Alternatively, reaction of **80** with H_2_S might directly lead to **1** without the need for formation of **81**. This reaction can be induced by an *O*-acetylserine lyase (CDIF630erm_01767) present in *C. difficile* (Gu et al., 2018b). Although H_2_S cannot be directly detected by our analytical method, its formation by *C. difficile* is well-known (Dubois et al., 2016; Gu et al., 2018b). Finally, instead of cysteine, thiocysteine might transfer one sulfur, as is the case in iron–sulfur-cluster biosynthesis (Freibert et al., 2021).

In addition, methionine **(82)** can release methanethiol **(83)** *via* methionine γ-lyase activity known from *C. difficile* 630Δ*erm* (CD630-35770-mdeA; Dubois et al., 2016). Methanethiol metabolism to di- and trisulfides has been described (Dickschat et al., 2010) and would lead to disulfide **18** by combination of **1** and **83**. In absence of cysteine, methionine may also act as a substitute sulfur source by generating homocysteine through SAM cycle (CDIF630erm_00247 and CDIF630erm_03920), which can either feed into the transsulfuration or release H_2_S. In addition to simple alcohol analogs of the amino acids valine, leucine, isoleucine, and alanine, several sulfides of longer or unsaturated alcohols occur in the volatile extracts. These might be formed from elongation processes and desaturations and are components of Stickland and butanoate fermentation. For example, Stickland fermentation of two leucine molecules will lead to 3-methylbutanoic and 4-methylpentanoic acids, the latter containing the carbon backbone of compounds **9**, **32**, and **47–49**.

The high disulfide concentration might hint toward a function of the sulfur VOCs in the oxidative stress response in *C. difficile*, in which involvement of a desulfhydrase, converting cysteine into sulfide, ammonia, and pyruvate has been shown (Dubois et al., 2016; Morvan et al., 2021). Although hypothetically, the generated sulfide may increase formation of the thiols discussed here. These thiols might function as a sort of movable protecting groups. Under access of oxygen they can form disulfide bonds to sensitive thiol centers groups, thus protecting them from further oxidation. This process might be reversible, depending on local thiol concentration.

### Specificity and Potential Biological Function of the Identified Volatiles

Numerous volatile compounds have been described associated with (Garner et al., 2007) or produced by *C. difficile* earlier (Rees et al., 2016). Out of 105 VOCs detected in this study, 14 have been previously reported. These include the sulfur compounds 1-butanethiol **(3)**, 3-methyl-1-butanethiol **(4)**, 3-methyl-2-butene-1-thiol **(6)**, 4-methyl-1-pentanethiol **(9)**, methyl butyl disulfide **(20)**, the *S*-methyl esters **51** and **53**, 2,2-dimethylthiirane **(52)**, trithiolane **(56)**, as well as five additional VOCs (**67**, **68**, **75**, **76**, and **77**; Rees et al., 2016). Nevertheless, the study of Rees et al. (2016) analyzed the volatiles indirectly, first isolating the supernatant from the cells by centrifugation and storage, followed by headspace analysis of the supernatant *via* SPME. In contrast, our analysis was performed on living cultures *via* adsorbents. This difference might explain the higher sulfur content in our analysis, underlining the importance of these compounds for *C. difficile*.

For 17 other compounds bacterial producers have been described. Among them are the common bacterial volatiles (Weisskopf et al., 2021) dimethyl disulfide **(14)** and dimethyl trisulfide **(54)**. 2-Methyl-1-propanethiol **(1)** is a compound produced by the pathogenic oral bacterium *Porphyromonas gingivalis* (Roslund et al., 2021). Disulfides **15–17** and bis(1-methylethyl) trisulfide **(58)** were found in *Phaeobacter gallaeciensis* and *Oceanibulbus indolifex* by feeding experiments (Dickschat et al., 2010). A comprehensive study by Citron et al. (2012) showed that 1,2,4-trithiolane **(55)**, sulfur **(64)**, 4-methyl-1-pentanol **(69)**, 1-hexanol **(70)**, 5-methylhexanol **(71)** and 6-methyl-5-heptene-2-one **(78)** were synthesized by different *Streptomyces* bacteria. 2-Methyl-1-propanol **(65)** is e.g., an important metabolite of *Mycobacterium bovis* (Rajanikanth et al., 1984) and a major target of biotransformations for biofuel production (Li et al., 2011). Butanol **(66)** and 2-methylpropanal **(72)** are common bacterial volatiles, e.g., reported from *Streptococcus pneumoniae* (Filipiak et al., 2012), while 2-Methylbutanal **(74)** was reported from *Mycobacterium avium* subsp. *paratuberculosis* (Trefz et al., 2013). Methylcyclopentane **(73)** is produced by stomach cancer associated bacterium *Helicobacter pylori* (Buszewski et al., 2008).

For thiols **2**, **5**, **7**, and **11**, disulfides **18**, **19**, **21–24**, **27**, **31**, **35**, **39**, and **41**, as well as trisulfides **59** and **60** producers of animal, plant, and fungal origins are known (Andersen et al., 1982; Wood, 1990; Noleau et al., 1991; Näf and Velluz, 1996; Cho et al., 2003; McLean et al., 2012; Karimi et al., 2020; Marcinkowska et al., 2021). To our knowledge, no natural producers were reported for thiols **8**, **10**, **12**, and **13**, disulfides **25**, **26**, **28–30**, **32–34**, **36–38**, **40**, and **42–50**, as well as trisulfides **61–63**. Hence, we propose these 28 compounds to be new natural products.

Few *C. difficile* 630Δ*erm* VOCs have been investigated for their various biological functions. Dimethyl disulfide **(14)** is a most prominent and common bacterial volatile (Dandurishvili et al., 2011; Huang et al., 2012; Bletz et al., 2019) for which both stimulating and inhibiting effects on bacterial growth have been shown (Dandurishvili et al., 2011; Garbeva et al., 2014; Popova et al., 2014). Further biological activities on fungi (Fernando et al., 2005; Popova et al., 2014), plants (Huang et al., 2012; Groenhagen et al., 2013; Meldau et al., 2013), and animals (Huang et al., 2010; Popova et al., 2014) were reported. Dimethyl trisulfide **(54)** inhibited growth of *Serratia marcescens*, *Staphylococcus aureus*, and *Escherichia coli* (Tyc et al., 2015). *Escherichia coli* is also affected by 1-butanol **(66)**, which is able to inhibit its biofilm formation (Létoffé et al., 2014). *S*-Methyl ethanethioate **(51)** is a growth inhibiting factor in bacteria-fungal interactions (Ossowicki et al., 2017).

### Variations in Cysteine and Methionine Supply Result in Metabolic Changes

The composition of VOCs of *C. difficile* 630Δ*erm* was strongly altered when varying amounts of sulfur-containing amino acids were used as substrates. This variation seems to be part of the metabolic adaptation process as adjustment to a changing surrounding.

As Dubois et al. (2016) showed, expression of genes involved in cysteine metabolism, amino acid biosynthesis, stress response, fermentation, energy metabolism, and iron uptake are influenced by cysteine. The authors also suggested that high cysteine concentrations in the growth medium mimics conditions of iron depletion by inducing expressions of the ferric uptake regulator (Fur), as well as several proteins responsible for iron transport. This response results in a decreased availability of Stickland and butanoate fermentation products such as butanoate, pentanoate (valerate), 4-methylpentanoate (isocaproate), and 5-methylhexanoate (Berges et al., 2018). Since these compounds most likely function as precursors to thiols, the decreased amounts of 1-butanethiol **(3)**, 1-pentanethiol **(7)**, 4-methyl-1-pentanethiol **(9)**, and 5-methyl-1-hexanethiol **(12)** found at high cysteine levels are in full accordance with the aforementioned studies. Cysteine is also efficiently degraded into sulfide and pyruvate by cysteine desulfidase (CD630_32320) operative at high cysteine levels (Gu et al., 2018b), but additionally other enzymes such as methionine γ-lyase, cystathionine-β-lyase, or *O*-acetylserine lyase might also be involved (Dubois et al., 2016; Gu et al., 2018b).

A second reason for the decreased amounts of thiols at high cysteine levels may be the upregulation of an unknown oxidizing enzyme that catalyzes the formation of disulfides, to counteract accumulation of thiols. Such an oxidizing enzyme may also protect physiologically important cysteines in proteins when oxidative stress is increasing. Figure 5 shows a lower total number of disulfides at high cysteine level but in most cases higher amounts for each disulfide. This may be a result of a protection mechanism against high concentrations of thiols.

A lower cysteine concentration in the medium resulted in lower amounts of thiols which confirms the central role of this amino acid in the formation of thiols. Disulfides were affected differently, as disulfides carrying more than five carbons were less abundant, while smaller disulfides dominated. Similar observations were made the CDMM+M medium. Disulfides carrying less than eight carbons were increased, while the remaining disulfide levels, as well as thiols remained either unchanged or decreased. This suggests a close relation between cysteine and methionine in regulation of the thiol and disulfide metabolism. Methanethiol may be released from methionine and then incorporated in the disulfide formation process, thus increasing the number of methyl alkyl disulfides. Moreover, insufficient cysteine supply may also be compensated by incorporation of methionine into the sulfur metabolism (Dubois et al., 2016; Gu et al., 2018b). Methionine would then be used to generate additional cysteine and would inevitably lead to an alteration of the VOC profile of thiols and disulfides.

In conclusion, we show here a detailed analysis of structures of *C. difficile* 630Δ*erm* released VOCs, which show a complex composition of mostly sulfur-containing volatiles. The availability of and balance between methionine and cysteine functioning as a sulfur source determined the constitution of volatile thiols and disulfides. High amounts of cysteine in the medium resulted in a less diverse set of volatiles caused by missing precursors. Low amounts of cysteine in the medium resulted in overall decreased amounts of thiols and disulfides, that is most likely alleviated by methionine. Increasing methionine concentration in the medium resulted in a concentration shift toward shorter disulfides. The results gave first insight into the structure and biosynthetic formation of *C. difficile* VOCs, although more detailed enzymatic and functional studies are needed to clarify the underlying biosynthetic pathways as well as the physiological and ecological effects of these unique compounds.

## Data Availability Statement

The original contributions presented in the study are included in the article/**Supplementary Material**, further inquiries can be directed to the corresponding author.

## Author Contributions

PB, MN-S, DJ, and SS conceived the idea. PB was responsible for the micro reactions, GC/MS analyses, and data analysis. MN-S and PH performed the cultivations and sampling. PB and MN-S were responsible for data integration and writing of the draft manuscript with input of all authors. All authors contributed to the article and approved the submitted version.

## Funding

This work was funded by the Federal State of Lower Saxony, Niedersächsisches Vorab CDiff and CDInfect projects (VWZN2889/3215/3266). Furthermore, grants for open access publication were made available by the TU Braunschweig central library.

## Conflict of Interest

The authors declare that the research was conducted in the absence of any commercial or financial relationships that could be construed as a potential conflict of interest.

## Publisher’s Note

All claims expressed in this article are solely those of the authors and do not necessarily represent those of their affiliated organizations, or those of the publisher, the editors and the reviewers. Any product that may be evaluated in this article, or claim that may be made by its manufacturer, is not guaranteed or endorsed by the publisher.
