# Supplementary material for: Thiol Metabolism and Volatile Metabolome of Clostridioides difficile
Source: Front Microbiol. 2022 Jun 16;13:864587. doi: 10.3389/fmicb.2022.864587 (PMC9243749; doi:10.3389/fmicb.2022.864587)
Supplement: Supplementary file 2 [file Data_Sheet_1.PDF]

Supplementary information

Caption for table Table S1. Volatile organic compounds found in headspace extracts of *Clostridioides difficile* 630Δ*erm* cultivated in four CDMM derived media. The different media are abbreviated as follows, **CDMM+M**: CDMM + 1.0 g/l methionine, **CDMM-C**: CDMM + 0.1 g/l cysteine, **CDMM**: CDMM + 0.5 g/l cysteine and **CDMM+C**: CDMM + 2.0 g/l cysteine. The molecular weight (MW) is given in Da. Compound identification was based on comparison of spectra with those of data bases and mass spectrometric fragmentation(ms), comparison of retention index to published values on the same or similar GC phases (ri), as well as comparison to commercially available or synthetic reference compounds (std). Retention indices of GC phases related to the HP5-MS phase used are marked with an asterisk. Retention indices from our own database are shown in italic. Compounds, that were not detected are marked with n.d.. The average integrated signals (AvgIS) were calculated from three replicates of integrated signals (IS1, IS2 and IS3). Fold changes (AvgIS FC) were calculated as ratio of the average integrated signal in the respective medium to the average integrated signal in CDMM. When a compound was absent in the reference medium, the lowest detected average integrated signal was used as referencing point. Reciprocal foldchanges are shown as AvgIS rFC. P-values calculated from Wilcoxon-Mann-Whitney test were obtained for each test medium (CDMM+M, CDMM-C and CDMM+C) against the reference medium (CDMM) and corrected with Benjamini-Hochberg method. Compounds that were not detected and data that could not be calculated are marked with N/A. Medium constituents are not shown.

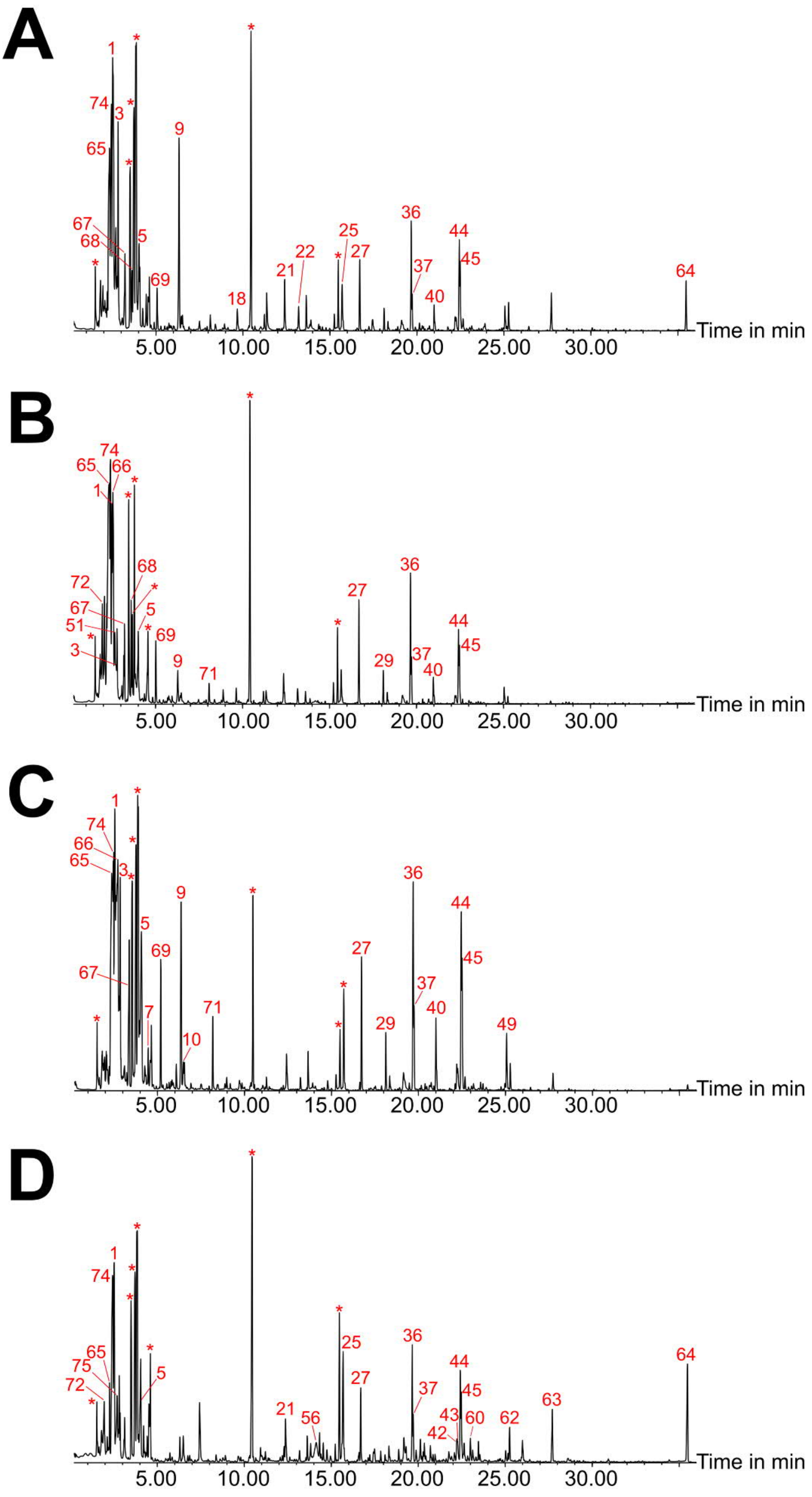

Figure S1. Representative total ion chromatograms of *Clostridioides difficile* 630Δ*erm*, cultivated in CDMM+M (**A**), CDMM-C (**B**), CDMM (**C**) and CDMM+C (**D**). Twenty compounds with highest intensities are shown for each medium. Media constituents and artefacts are marked with asterisk.

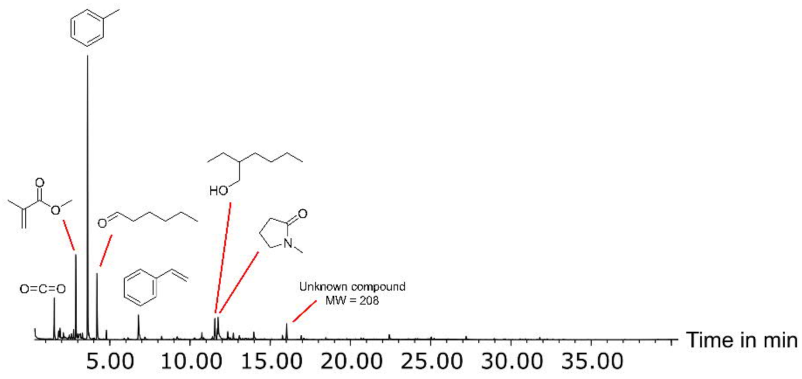

Figure S2. Total ion chromatogram of the unoculated medium.

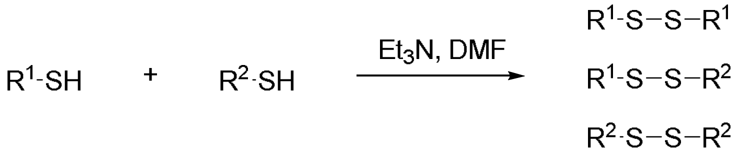

Figure S3. Combinatorial synthesis of disulfide mixtures.

Table S2. Synthesized disulfides for the identification of volatile disulfides produced by *Clostridioides difficile* 630Δ*erm*. Molecular weights (MW; in Da), gas chromatographic retention indices (RI) on a HP5-MS phase and important EI-MS fragments (70 eV) are shown.

Name	Formula	MW	*RI*	EI-MS: *m/z* (%)
1-Methylethyl 2-methylbutyl disulfide	C8H18S2	178	1223	178 (70), 136 (14), 108 (24), 71 (82), 55 (13), 43 (100), 41 (34), 39 (16)
1-Methylethyl 3-methylbutyl disulfide	C8H18S2	178	1225	178 (86), 136 (48), 103 (16), 71 (68), 70 (11), 55 (15), 43 (100), 41 (34), 39 (14)
1-Methylethyl pentyl disulfide	C8H18S2	178	1268	178 (77), 136 (42), 108 (12), 103 (27), 71 (62), 43 (100), 41 (41), 39 (14)
1-Methylethyl propyl disulfide	C6H14S2	150	1068	150 (75), 108 (65), 66 (11), 43 (100), 41 (39), 39 (15)
2-Methylbutyl 2-methylpropyl disulfide	C9H20S2	192	1316	192 (84), 122 (23), 71 (83), 57 (100), 56 (10), 55 (19), 43 (82), 41 (54), 39 (15)
2-Methylbutyl 3-methyl-2-butyl disulfide	C10H22S2	206	1394	206 (26), 136 (12), 71 (100), 55 (16), 43 (65), 41 (19)
2-Methylbutyl 3-methylbutyl disulfide	C10H22S2	206	1424	206 (57), 136 (38), 71 (100), 55 (15), 43 (91), 41 (27)
2-Methylbutyl pentyl disulfide	C10H22S2	206	1467	206 (62), 136 (32), 103 (14), 71 (100), 70 (11), 55 (19), 43 (92), 41 (34), 39 (11)
2-Methylbutyl propyl disulfide	C8H18S2	178	1268	178 (70), 108 (45), 71 (47), 55 (15), 43 (100), 41 (34), 39 (13)
2-Methylpropyl 2-propyl disulfide	C7H16S2	164	1117	164 (61), 122 (21), 108 (16), 57 (100), 43 (40), 41 (45), 39 (14)
2-Methylpropyl pentyl disulfide	C9H20S2	192	1361	192 (67), 136 (24), 103 (12), 71 (30), 57 (100), 55 (17), 43 (45), 42 (11), 41 (47), 39 (16)
2-Methylpropyl propyl disulfide	C7H16S2	164	1161	164 (99), 108 (52), 73 (11), 57 (100), 47 (10), 45 (12), 43 (58), 41 (66), 39 (18)
3-Methyl-2-butyl 1-methylethyl disulfide	C8H18S2	178	1193	178 (31), 108 (23), 71 (78), 55 (14), 43 (100), 41 (26)
3-Methyl-2-butyl 2-methylpropyl disulfide	C9H20S2	192	1288	192 (33), 122 (16), 71 (100), 57 (34), 55 (20), 43 (85), 41 (30), 39 (12)
3-Methyl-2-butyl pentyl disulfide	C10H22S2	206	1441	206 (25), 136 (11), 71 (100), 55 (14), 43 (71), 41 (20), 39 (11)
3-Methyl-2-butyl propyl disulfide	C8H18S2	178	1241	178 (30), 108 (17), 71 (77), 55 (14), 43 (100), 41 (24), 39 (10)
3-Methylbutyl 2-methylpropyl disulfide	C9H20S2	192	1318	192 (80), 136 (28), 87 (11), 71 (49), 70 (10), 57 (100), 55 (22), 43 (64), 41 (52), 39 (15)
3-Methylbutyl 3-methyl-2-butyl disulfide	C10H22S2	206	1397	206 (23), 136 (16), 71 (100), 55 (17), 43 (79), 41 (22)
3-Methylbutyl pentyl disulfide	C10H22S2	206	1468	206 (78), 136 (34), 103 (14), 71 (79), 70 (13), 69 (10), 55 (21), 43 (100), 41 (33), 39 (13)
3-Methylbutyl propyl disulfide	C8H18S2	178	1269	178 (76), 108 (21), 71 (49), 55 (15), 43 (100), 41 (33), 39 (11)
Bis(1-methylethyl) disulfide	C6H14S2	150	1021	150 (78), 108 (69), 66 (15), 59 (11), 43 (100), 41 (34), 39 (14)
Bis(2-methylbutyl) disulfide	C10H22S2	206	1422	206 (53), 136 (19), 71 (100), 70 (10), 55 (18), 43 (81), 41 (29)
Bis(2-methylpropyl) disulfide	C8H18S2	178	1209	178 (47), 122 (15), 57 (100), 55 (10), 43 (11), 41 (40), 39 (12)
Bis(3-methyl-2-butyl) disulfide	C10H22S2	206	1362	206 (24), 136 (10), 71 (100), 55 (14), 43 (54), 41 (15)
Bis(3-methylbutyl) disulfide	C10H22S2	206	1425	206 (71), 136 (17), 102 (10), 71 (81), 70 (12), 55 (24), 43 (100), 41 (26), 39 (10)

Butyl (1-methylethyl) disulfide	C7H16S2	164	1166	164 (100), 122 (69), 57 (100), 45 (10), 43 (51), 41 (54), 39 (17)
Butyl 2-methylbutyl disulfide	C9H20S2	192	1366	192 (91), 122 (55), 71 (82), 57 (54), 55 (20), 43 (100), 41 (45), 39 (13)
Butyl 2-methylpropyl disulfide	C8H18S2	178	1259	178 (52), 122 (27), 57 (100), 41 (37)
Butyl 3-methyl-2-butyl disulfide	C9H20S2	192	1340	192 (31), 122 (20), 71 (100), 57 (23), 55 (19), 43 (82), 41 (24), 39 (10)
Butyl 3-methylbutyl disulfide	C9H20S2	192	1367	192 (100), 136 (15), 122 (20), 87 (14), 71 (75), 57 (38), 55 (25), 43 (85), 41 (40), 39 (13)
Butyl hexyl disulfide	C10H22S2	206	1512	206 (100), 150 (17), 122 (77), 85 (32), 57 (79), 56 (19), 55 (28), 43 (87), 41 (55)
Butyl pentyl disulfide	C9H20S2	192	1409	192 (100), 136 (18), 122 (54), 103 (13), 71 (39), 57 (60), 55 (18), 43 (69), 41 (52), 39 (12)
Butyl propyl disulfide	C7H16S2	164	1208	164 (100), 122 (14), 108 (53), 57 (59), 47 (12), 43 (60), 41 (59), 39 (16)
Dibutyl disulfide	C8H18S2	178	1308	178 (74), 122 (49), 90 (11), 87 (11), 57 (100), 56 (15), 55 (14), 47 (11), 45 (10), 41 (48)
Diethyl disulfide	C4H10S2	122	927	122 (100), 94 (48), 66 (53), 41 (48)
Dihexyl disulfide	C12H26S2	234	1715	234 (61), 150 (45), 117 (24), 85 (48), 57 (19), 56 (15), 55 (26), 43 (100), 41 (33)
Dipentyl disulfide	C10H22S2	206	1510	206 (78), 136 (50), 103 (26), 71 (66), 69 (12), 55 (17), 43 (100), 42 (15), 41 (35)
Dipropyl disulfide	C6H14S2	150	1109	150 (99), 108 (53), 66 (14), 45 (10), 43 (100), 41 (37), 39 (15)
Ethyl 1-methylethyl disulfide	C5H12S2	136	977	136 (74), 96 (10), 94 (100), 66 (36), 59 (12), 43 (36), 41 (26)
Ethyl 2-methylbutyl disulfide	C7H16S2	164	1177	164 (87), 94 (63), 71 (47), 66 (12), 55 (19), 45 (10), 43 (100), 41 (31), 39 (12)
Ethyl 2-methylpropyl disulfide	C6H14S2	150	1070	150 (100), 94 (76), 79 (11), 66 (18), 57 (87), 55 (12), 45 (10), 43 (10), 41 (54), 39 (14)
Ethyl 3-methyl-2-butyl disulfide	C7H16S2	164	1152	164 (35), 94 (21), 71 (85), 61 (10), 59 (13), 55 (22), 43 (100), 41 (20), 39 (11)
Ethyl 3-methylbutyl disulfide	C7H16S2	164	1178	164 (93), 94 (33), 71 (35), 55 (26), 43 (100), 41 (22)
Ethyl butyl disulfide	C6H14S2	150	1117	150 (97), 96 (10), 94 (100), 66 (24), 57 (32), 45 (10), 41 (44), 39 (10)
Ethyl hexyl disulfide	C8H18S2	178	1324	178 (85), 94 (84), 57 (17), 56 (10), 55 (17), 43 (100), 41 (30)
Ethyl pentyl disulfide	C7H16S2	164	1221	164 (100), 94 (98), 43 (90)
Ethyl propyl disulfide	C5H12S2	136	1017	136 (100), 94 (90), 66 (37), 45 (10), 43 (39), 41 (29), 39 (10)
Hexyl 1-methylethyl disulfide	C9H20S2	192	1371	192 (61), 150 (37), 117 (23), 108 (14), 85 (43), 57 (14), 55 (13), 43 (100), 41 (32), 39 (12)
Hexyl 2-methylbutyl disulfide	C11H24S2	220	1569	220 (64), 150 (27), 117 (13), 85 (24), 71 (83), 57 (12), 55 (24), 43 (100), 41 (34)
Hexyl 2-methylpropyl disulfide	C10H22S2	206	1464	206 (61), 150 (18), 117 (13), 85 (23), 57 (100), 56 (15), 55 (20), 43 (48), 41 (47), 39 (12)
Hexyl 3-methyl-2-butyl disulfide	C11H24S2	220	1543	220 (26), 150 (13), 85 (10), 71 (100), 55 (17), 43 (79), 41 (23)
Hexyl 3-methylbutyl disulfide	C11H24S2	220	1570	220 (69), 136 (29), 85 (13), 71 (61), 55 (22), 43 (100), 41 (30)
Hexyl pentyl disulfide	C11H24S2	220	1613	220 (75), 150 (18), 136 (34), 117 (15), 103 (13), 85 (29), 71 (33), 55 (20), 43 (100), 41 (34)
Hexyl propyl disulfide	C9H20S2	192	1415	192 (70), 108 (60), 85 (14), 57 (13), 55 (18), 43 (100), 41 (38)
Pentyl propyl disulfide	C8H18S2	178	1311	178 (76), 108 (59), 71 (22), 55 (11), 45 (10), 43 (100), 41 (33), 39 (13)
